# lncRNA MIR22HG-Derived miR-22-5p Enhances the Radiosensitivity of Hepatocellular Carcinoma by Increasing Histone Acetylation Through the Inhibition of HDAC2 Activity

**DOI:** 10.3389/fonc.2021.572585

**Published:** 2021-02-24

**Authors:** Qiao Jin, Hao Hu, Siqi Yan, Long Jin, Yuliang Pan, Xiangjun Li, Yayi Peng, Peiguo Cao

**Affiliations:** ^1^ Department of Oncology, Third Xiangya Hospital of Central South University, Changsha, China; ^2^ Department of Oncological Radiotherapy, Hunan Academy of Traditional Chinese Medicine Affiliated Hospital, Changsha, China; ^3^ Department of Oncology, The Second People’s Hospital of Hunan Province, Changsha, China

**Keywords:** MIR22HG, miR-22-5p, radiosensitivity, hepatocellular carcinoma, HDAC, histone (de)acetylation

## Abstract

**Background:**

With the development of radiotherapy technology, radiotherapy has been increasingly used to treat primary hepatocellular carcinoma (HCC). However, due to radioresistance and the intolerance of the adjacent organs to radiation, the effects of radiotherapy are often unsatisfactory. Therefore, it is necessary to study radiosensitization in HCC.

**Method:**

A microarray was used to analyze the genes that were significantly associated with radiosensitivity. HCC cells, HepG2 and MHCC97H, were subjected to radiation *in vitro*. Real-time PCR was performed to determine MIR22HG (microRNA22 host gene) and miR-22-5p expression levels. Western blotting was performed to determine histone expression levels. A histone deacetylase (HDAC) whole cell assay was used to determine the activity of HDAC2. MTT, colony formation, 5-ethynyl-2′-deoxyuridine, and wound healing assays were performed to examine the function of MIR22HG and miR-22-5p in cellular radiosensitivity. Chromatin immunoprecipitation-PCR was used to confirm that HDAC2 affects the acetylation level of the MIR22HG promoter region. Finally, animal experiments were performed to demonstrate the *in vivo* effect of MIR22HG on the radiosensitivity of hepatoma.

**Results:**

Irradiation can up-regulate MIR22HG expression and down-regulate HDAC2 expression. Inhibition of HDAC2 expression promotes histone acetylation in the MIR22HG promoter region and up-regulates MIR22HG expression. MIR22HG can increase radiosensitivity *via* miR-22-5p in HCC.

**Conclusion:**

Inhibition of HDAC2 expression promotes histone acetylation in the MIR22HG promoter region, thereby up-regulating the expression of MIR22HG and promoting the production of miR-22-5p, and ultimately increasing the sensitivity of liver cancer radiotherapy.

## Introduction

Primary liver cancer (hepatocellular carcinoma; HCC) is one of the most common malignant cancers worldwide, and ranks third for cancer-related mortality (1, 2). Owing to the lack of specific symptoms in the early stage of liver cancer, patients are often in the late stage of HCC at the time of treatment. The standard treatment for HCC depends on a variety of factors, including the tumor stage, basic liver function, and general condition of the patient. The main treatments include surgery, chemotherapy, and radiotherapy (3, 4). HCC is moderately sensitive to radiation, and the sensitivity is equivalent to the radiosensitivity demonstrated by poorly differentiated squamous cell carcinoma (5). With the development of radiotherapy technology, increasingly precise radiotherapy techniques have emerged, such as three-dimensional conformal radiotherapy, stereotactic radiotherapy, and image-guided radiation therapy. The status of radiotherapy in the comprehensive treatment of liver cancer has also received increasing attention (6, 7). Radiotherapy effectively prolongs the survival rate of patients with unresectable liver cancer (8–10). However, due to the presence of surrounding tissues and organs such as normal liver tissue, small intestines, and kidneys, it is often not possible to administer an adequate therapeutic dose. Therefore, achieving curative effects in a lower dosage range and effectively protecting the surrounding vital organs is a clinical challenge that warrants immediate attention. Identifying new radiosensitizing targets for liver cancer is likely to provide more powerful treatment strategies for liver cancer radiotherapy.

Long non-coding RNAs (lncRNAs) are RNAs longer than 200 nucleotides with no or a limited ability to encode proteins (11). They can promote tumor proliferation, invasion, migration, and radioresistance through various mechanisms that affect tumor development (12–14). lncRNAs mainly play a role in the regulation of the expression levels of genes through the sponge adsorption of miRNA. lncRNAs directly bind to proteins to regulate the stability of bound proteins (15). lncRNAs can affect the radiosensitivity of liver cancer; however, this specific mechanism requires further clarification. Previously, using microarray analysis, we found that MIR22HG expression level was moderately up-regulated after the HCC cell type MHCC97H was irradiated. MIR22HG plays a role as a tumor suppressor gene in liver (16), lung (17), and endometrial (18) cancers. Silencing MIR22HG can increase the proliferation, metastasis, and invasion of tumor cells (16–18). However, studies regarding the sensitivity of MIR22HG to radiotherapy for HCC have not yet been reported.

The location on the chromosome and the similarity of the sequences indicate that some lncRNAs may be the maternal gene of miRNA, and the expression of this type of lncRNA is closely related to the expression of miRNA (19). For example, the host gene of miR-100, MIR100HG, can produce miR-100 and miR-125b, which are involved in cetuximab sensitivity regulation (20). Although lncRNAs can be used as miRNA host genes, in some cases, they act independently of each other, as in the case of miR-1207-5p and its host gene in diabetic nephropathy (21). MIR22HG acts as a tumor suppressor by producing miR-22 (16). However, the function of MIR22HG and miR-22-5p in HCC radiotherapy has not yet been reported.

Histone deacetylases (HDAC) are a class of histone-modifying enzymes that play an important role in the structural modification of chromosomes and regulation of gene expression (22). HDAC deacetylates the histones and binds tightly to the negatively charged DNA. The chromatin is tightly curled and transcription of the gene is inhibited. Under appropriate conditions, the acetylation/deacetylation of the promoter region of lncRNA ultimately affects the expression level of the gene (23). In this study, we found that the expression level of MIR22HG was moderately up-regulated after the HCC cell type MHCC97H was irradiated. In contrast, the expression level of HDAC2 showed a downward trend after HCC cells were irradiated. The inhibition of HDAC2 activity increases histone acetylation of the MIR22HG promoter region, thereby up-regulating the expression of MIR22HG and miR-22-5p and increasing the radiosensitivity of HCC cells. Therefore, our study provides a novel understanding of lncRNA and radiosensitivity in HCC and suggests lncRNAs as potential cancer biomarkers and therapeutic targets.

## Methods and Materials

### Cell Lines and Transfection

The hepatoma cell lines, Huh7, HepG2, Hep3B, BEL-7402, MHCC97H, and MHCC97L as well as human normal liver cell line L02 were purchased from the American Type Culture Collection (ATCC). Cells were cultured in Dulbecco’s modified Eagle’s medium (DMEM) supplemented with 10% fetal bovine serum (FBS; Gibco, NY, USA), 100 U/ml penicillin, and 100 μg/ml streptomycin, and incubated in a 5% CO_2_ atmosphere at 37°C. microRNA miR-22-5p inhibitors, lncRNA MIR22HG overexpression plasmid, small nuclear RNA against lncRNA MIR22HG, and negative control (NC) (GeneChem Biotechnology Company, Shanghai, China) were used to transfect the cells. First, the cells were seeded in a six-well culture plate and, after 24 h of cultivation, the degree of cell fusion reached more than 70%. Subsequently, according to the manufacturer’s instructions, the cells were transfected with Lipofectamine 3000 (Thermo Fisher Scientific).

### Quantitative Real-Time PCR (qPCR)

PCR was used to detect the mRNA expression level. Total RNA was extracted from the above cells using TRIzol Reagent (Invitrogen Life Technologies). Subsequently, a First Strand cDNA Synthesis Kit (Fermentas, Vilnius, Lithuania) was used to synthesize cDNA from total RNA using random primers. *U6* and *GAPDH* were used as housekeeping controls. The forward and reverse primer sequences were as follows: MIR22HG, 5′-CAGGAGCCTGTTCCTCTCAC-3′ and 5′-GCCCAAAACGTATCATCCAC-3′; GAPDH, 5′-GGAGCGAGATCCCTCCAAAAT-3′ and 5′-GGCTGTTGTCATACTTCTCATGG-3′; miR-22-5p, 5′-ACACTCCAGCTGGGAGTTCTTCAGTGGCAA-3′ and 5′-CTCAACTGGTGTCGTGGAGTCGGCAATTCAGTTGAGTAAAGCTT-3′; U6, 5′-CTCGCTTCGGCAGCACA-3′ and 5′-AACGCTTCACGAATTTGCGT-3′.

Real-time PCR was performed with a 7300 Fast Real-time PCR system (Applied Biosystems, NY, USA) using a SYBR Green qPCR Mix (TOYOBO). Relative quantification was calculated according to the Δ Δ CT method.

### X-ray Irradiation

The experimental group was irradiated with an X-ray generator (Varian, USA). In the irradiation process, six dose gradients of 0, 1, 2, 4, and 6 Gy were set.

### Histone Acetyltransferase Inhibitor and Histone Deacetylase Inhibitor

The histone acetyltransferase inhibitor uses C646 with a concentration gradient of 5, 10, 15, and 20 µM, and the optimal concentration was selected. Related doses for HDAC inhibitors using Santacruzamate A have been reported in previous studies (24).

### MTT Assay

HepG2 and MHCC97H cells were cultured in 96-well cell culture plates at 5 × 10^3^ cells/well and were incubated in 100 µl of DMEM for 72 h after irradiation with 4 Gy. Following this, 50 μl of 3-(4,5-dimethyl-2-thiazolyl)-2,5-diphenyl-2-hzozolium (Sigma Chemicals) and 5 mg/ml of PBS were added to each well. Cells were then incubated for 4 h. Finally, 150 μl of dimethyl sulfoxide was added to each well to dissolve the crystals, and the optical density of each culture well was measured at a wavelength of 570 nm.

### Wound Healing Assay

Approximately 1 × 10^5^ cells/ml after transfection and irradiation (4 Gy) were plated in six-well plates. The cell coverage of the plates was observed until the cells reached 90% confluency. Following this, a pipette was used to draw a scratch with a width of 1 mM on the bottom of the plate covered with cells, and the separated cells were aspirated. After the scratches were completed, the medium containing 10% FBS was replaced with a medium with a low FBS concentration, and an image was obtained by microscope (Motic, China) at 0 h as a control. After completing the above steps, the cells were placed in an incubator for 48 h and images were again obtained.

### Colony Formation Assay

The cells were seeded in a six-well culture plate at a density of 1,000 cells/well and treated with Santacruzamate A, transfected, and irradiated (4 Gy), and the culture plate was then placed in an incubator and incubated for 2 to 3 weeks. After the culture was completed, the cell colonies were fixed with 1 ml of 4% paraformaldehyde and stained with hematoxylin. If the number of cloned cells summed to 10, it was considered a clone.

### 5-ethynyl-2′-deoxyuridine (EdU) Assay

HepG2 and MHCC97H cells were seeded in 96-well culture plates at 5 × 10^3^ cells/well and transfected and irradiated according to the above experimental procedures. The cells were fixed with 2% paraformaldehyde, followed by EdU detection according to the instructions of the EdU kit (Click-iT^®^ Plus EdU Alexa-647^®^ Imaging Kit, Life Technologies), and imaged using a Nikon A1R-MP confocal microscope. After completing the experimental steps, EdU+ cells were quantified using ImageJ software.

### Western Blotting (WB)

Cells were washed with PBS and lysed with RIPA lysis buffer for 30 min. Protein concentrations were determined using a BCA Protein Assay Reagent kit (Thermo Scientific). Proteins were separated on 10% sodium dodecyl sulphate-polyacrylamide gel electrophoresis (SDS-PAGE) gels, blotted onto nitrocellulose membranes, and probed with anti-histone H3K4ac (1:1,000 Abcam, ab232931); anti-histone H3K9ac (1:5,000 Abcam, ab4441); anti-histone H3K27ac (1:200 Abcam, ab177178), anti-histone H3K23ac (1:500 Abcam, ab61234), anti-histone H3 (1:1,000 SANTA, sc-56616); and anti-GAPDH (1:2,000 Abcam, ab125247), followed by the appropriate horseradish peroxidase-conjugated secondary antibodies (Auragene, Changsha, China). Subsequently, immunodetection was performed using an ECL plus WB detection system (Auragene, Changsha, China). IPP 6.0 software was used to determine the signal strength. In the WB experiment, anti-β-actin antibody (1:500) was selected for immunoblotting as an internal control.

### Chromatin Immunoprecipitation (ChIP)-PCR

We conducted a ChIP study according to the steps reported in a previous study (25), collecting approximately 1.5 × 10^6^ cells and cross-linking them. Briefly, the immunoprecipitated protein-DNA complex was first obtained using anti-histone H3K4ac (1:1,000 Abcam, ab232931). Following this, a CFX96 Quantitative Thermal Cycler and SsoFast EvaGreen Low-ROX qPCR SuperMix were used to analyze the purified DNA. In the subsequent qPCR quantitative experiments, the primer sequences used were as follows (forward and reverse): MIR22HG, 5′-TTGGTCTCCACCTTCGAATC-3′ and 5′-AGAGCCTCCCAAGAGGTAGC-3′; GAPDH, 5′-TACTAGCGGTTTTACGGGCG-3′ and 5′-TCGAACAGGAGGAGCAGAGAGCGA-3′. Primers were designed based on the MIR22HG promoter regions.

### HDAC Whole-Cell Assay

Before starting the test, the cells were transfected or irradiated according to the previously described experimental steps. The HDAC activity of the cells and tissues were measured according to the instructions of the purchased HDAC fluorescence activity assay kit.

### Xenograft Mouse Model

All animal experiments were approved by the Animal Centre of Central South University and performed according to the International Guidelines and Protocols. First, 18–22 g, 5-week-old, female, nude-mice were purchased from the Shanghai Laboratory Animal Centre (SLAC, Shanghai, China). Cells (5 × 10^6^) were injected subcutaneously into the right abdomen of the nude mice. When the tumor volume increased to 10 mm^3^, the tumor was irradiated with a single dose of 4 Gy. The volume of the transplanted tumor was measured every 2 days, and the nude mice were euthanized after 25 days. The transplanted tumor was stripped, and the volume of the transplanted tumor was measured using Vernier calipers. The formula is as follows:

$$\text{Tumor volume}\;({\text{mm}}^{3})=(\text{longest diameter}\times {\text{shortest diameter}}^{2})/2$$

### Immunohistochemistry (IHC)

IHC staining was performed following the methods of previously published studies. Cancer tissue sections were de-paraffinized and rehydrated with graded alcohols, treated with 0.3% H_2_O_2_ in methanol for 30 min, and blocked with 1% PBA for 1 h. The prepared sections were incubated with Ki67 (Genetex, GTX16667) at 4°C overnight. The polymer enhancers were incubated at room temperature for 20 min and biotin-labeled secondary antibody was added and incubated at room temperature for 30 min. Next, the sections were stained with a DAB staining solution, counterstained with hematoxylin, then mounted in glycerol-vinyl-alcohol (GVA mount, Zymed). Two independent pathologists assessed the IHC scores in a blinded manner, and the details of the scoring methodology have been published previously (26).

### Bioinformatics Analysis

The ChIPbase (rna.sysu.edu.cn/chipbase/) was used to analyze the correlation between MIR22HG and miR-22-5p. The Oncolnc (http://www.oncolnc.org/) was used to extract TCGA database data for survival analysis.

### Expression Profile Analysis of lncRNAs and mRNAs

RNA expression profiling was performed using an Agilent human lncRNA microarray and mRNA microarray V.2.0 platform (GPL18109; Agilent Technologies, Inc., Santa Clara, CA, USA). Quantile normaliation and subsequent data processing were performed using Agilent Gene Spring Software 11.5 (Agilent Technologies, Inc.). Heatmaps representing differentially regulated genes were generated using Funrich 3.1.3 software.

### Statistical Analysis

GraphPad Prism 8.04 was used for all statistical analysis, and all experiments were repeated three times and expressed as mean ± SD. A significance test was performed on the dataset through analysis of variance. A T-test was used for comparisons between specific groups, and Tukey’s and Bonferroni tests were used for multiple comparisons between groups to analyze significance. The survival curve was drawn according to the Kaplan-Meier method and compared by log rank test. Cox regression analysis was used to analyze and evaluate survival data. A *P* value <0.05 was considered statistically significant.

## Results

### MIR22HG and miR-22-5p Are Down-regulated in HCC Tissues, and Radiotherapy Can Increase the Expression Level of miR-22-5p

**Figure 1:**
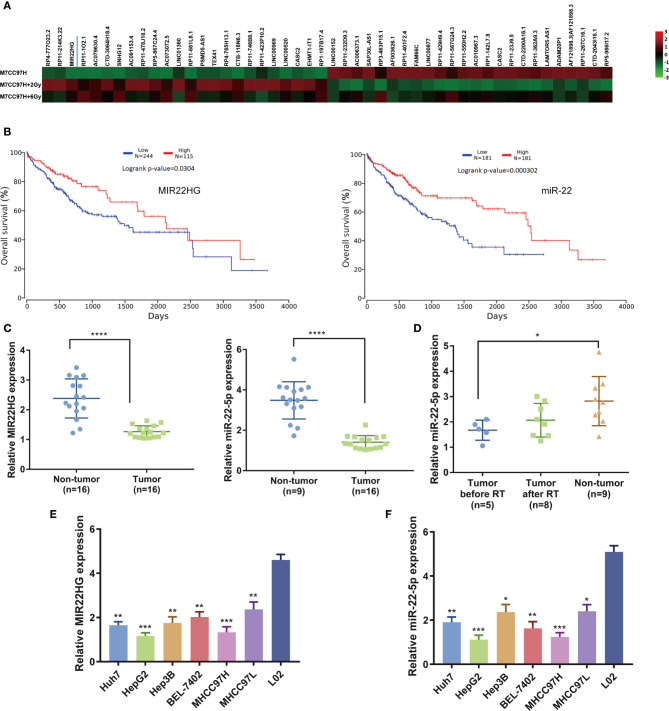
**(A)** Microarray results indicating that MIR22HG expression in MHCC97H cells increased significantly after irradiation. **(B)** Kaplan-Meier curves of the overall survival. The *P*-value was computed using the log-rank test. **(C)** mRNA expression of *MIR22HG* and miR-22-5p in tumor (n = 16) and non-tumor (n = 16) tissue. **(D)** miR-22-5p expression in blood samples of healthy individuals and patients with HCC before and after radiotherapy. **(E, F)** mRNA expression of *MIR22HG* and miR-22-5p in HCC cells Huh7, HepG2, Hep3B, BEL-7402, MHCC97H, and MHCC97L and in the human normal liver cell L02. *Compared with the L02 group. Mean ± SD (n = 3 independent experiments). *p < 0.05, **p < 0.01, ***p < 0.001, ****p < 0.0001.

Our previous microarray analysis showed that the expression level of MIR22HG was moderately up-regulated after hepatoma cells were irradiated compared to that of a control group (**Figure 1A**). TCGA database analysis found that the overall survival rate of patients with high expressions of MIR22HG and miR-22-5p was significantly higher than that of patients with low expressions of MIR22HG and miR-22-5p (**Figure 1B**). We examined the expression levels of MIR22HG and miR-22-5p in 16 pairs of HCC and adjacent tissues. Results showed that the expression levels of MIR22HG and miR-22-5p in adjacent tissues were higher than those in HCC tissues (**Figure 1C** **)**. Blood samples from 16 healthy individuals and 16 patients with HCC were retrieved before and after radiotherapy. Results showed that MIR22HG expression was not detected in blood samples, whereas miR-22-5p expression in the blood of healthy individuals was higher than that of patients with tumors. In patients, the level of blood miR-22-5p expression in after radiotherapy showed an increasing trend compared to that in patients before radiotherapy; however, this increase was not significant (*P* > 0.05) (**Figure 1D** **)**. These results indicate that the expression of MIR22HG and miR-22-5p decreased in hepatocarcinoma tissues and was positively correlated with prognosis. The miR-22-5p expression in the blood of patients with HCC showed an increasing trend after radiotherapy, indicating that MIR22HG and miR-22-5p may be tumor suppressors that are closely related to radiosensitivity in HCC. The PCR results showed that the expression levels of MIR22HG and miR-22-5p in six hepatocellular carcinoma cell lines were significantly lower than those in normal human liver cells L02 (**Figures 1E, F** **)**. Under the premise that the differential expression was significant, the expression level of MIR22HG in HepG2 and MHCC97H cells was the lowest among the six hepatocellular carcinoma cells (**Figures 1E, F** **)**. Therefore, we selected HepG2 and MHCC97H for further study.

### Irradiation Up-regulates MIR22HG Expression by Inhibiting HDAC2 Activity and Increasing Histone Acetylation of the MIR22HG Promoter Region

**Figure 2:**
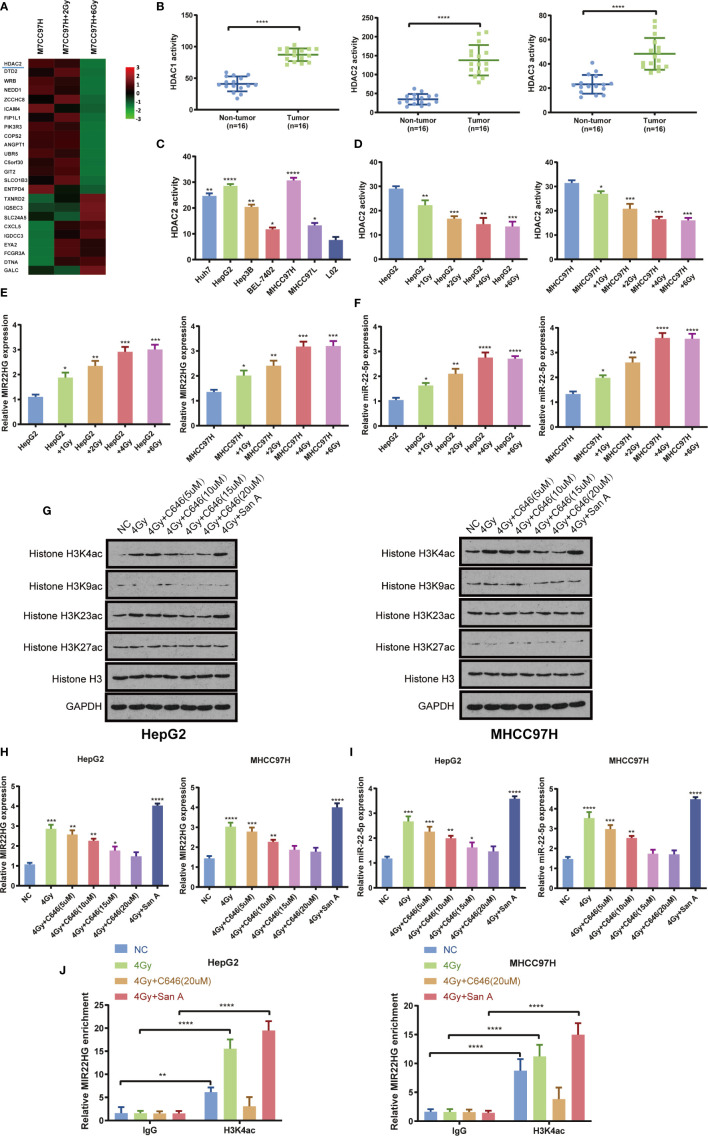
**(A)** Microarray results indicating that HDAC2 expression in MHCC97H cells decreased significantly after irradiation. **(B)** HDAC1, HDAC2, and HDAC3 activity in tumor (n = 16) and non-tumor (n = 16) tissues. **(C)** The activity of HDAC2 in the HCC cells Huh7, HepG2, Hep3B, BEL-7402, MHCC97H, and MHCC97L and the human normal liver cells L02. *Compared with the L02 group. **(D)** The activity of HDAC2 in HepG2 and MHCC97H cells treated with different doses of irradiation. * Compared with the HepG2 and MHCC97H groups. **(E, F)** The mRNA expression of *MIR22HG* and miR-22-5p in HepG2 and MHCC97H cells treated with different doses of irradiation (1, 2, 4, and 6 Gy). *Compared with HepG2 and MHCC97H groups. **(G)** Western blot analysis of histone H3K4ac, H3K9ac, H3K23ac, H3k27ac, and H3 in HepG2 and MHCC97H cells. The treatments were irradiation of 4 Gy alone, irradiation of 4 Gy + histone acetyltransferase inhibitor C646, irradiation of 4 Gy + histone deacetylation inhibitor Santacruzamate A. **(H, I)** Expression of MIR22HG and miR-22-5p in HepG2 and MHCC97H cells. The treatments were irradiation 4 Gy alone, irradiation of 4 Gy + histone acetyltransferase inhibitor C646, irradiation of 4Gy + histone deacetylation inhibitor Santacruzamate A. * Compared with the NC group. **(J)** ChIP-PCR showing the amplified signal of the MIR22HG promoter region. The treatments were irradiation of 4 Gy alone, irradiation of 4 Gy + histone acetyltransferase inhibitor C646, and irradiation of 4 Gy + histone deacetylation inhibitor Santacruzamate A. Results showed that both irradiation and drug intervention in HDAC2 can alter amplified signals of the MIR22HG promoter region. Mean ± SD (n = 3 independent experiments). *p < 0.05, **p < 0.01, ***p < 0.001, ****p < 0.0001. San A, Santacruzamate A.

Previous microarray analysis showed that the expression level of HDAC2 in the HCC cell MHCC97H radiation treatment group was lower than that in the control group (**Figure 2A** **)**. The activity of HDAC1, HDAC2, and HDAC3 was detected in 16 pairs of HCC and adjacent tissues. The results showed that the activities of HDAC1, HDAC2, and HDAC3 in HCC tissues were significantly higher than those in adjacent tissues (**Figure 2B** **).** The results showed that the activities of HDAC2, HDAC1, and HDAC3 in HepG2 and MHCC97H cells were higher than those in other cells (**Figure 2C**, **Supplementary Figures 1A, B** **)**. The cells were treated with different doses of radiation in HepG2 and MHCC97H, and the activity of HDAC2, HDAC1, and HDAC3 and the expression levels of MIR22HG and miR-22-5p were evaluated. Results showed that the activity of HDAC2 and the expression levels of MIR22HG and miR-22-5p showed the most obvious changes when cells were treated with 4 Gy of radiation (**Figures 2D–F**). This indicates that appropriate doses of radiation can inhibit the activity of HDAC2 and further demonstrates that radiation can up-regulate the expression levels of MIR22HG and miR-22-5p. Interestingly, the activities of HDAC1 and HDAC3 do not seem to be affected by radiation. There was no significant change in the activities of HDAC1 and HDAC3 in each group of HepG2 and MHCC97H (**Supplementary Figures 1C, D** **)**. We speculate that the activity of HDAC1 and HDAC3 may be related to other phenotypes of liver cancer. Combined with the results of the microarray, HDAC2 was selected for further study. WB showed that radiation increased histone acetylation, whereas acetyltransferase inhibitor C646 down-regulated histone-induced acetylation. Santacruzamate A, a deacetylase inhibitor, can up-regulate radiation-induced histone acetylation, with H3K4ac showing the most notable changes (**Figure 2G**). PCR results showed that radiation increased the expression levels of MIR22HG and miR-22-5p by up-regulating histone acetylation levels (**Figures 2H, I** **)**. ChIP-PCR results showed that the radioactivity induced histone acetylation of the MIR22HG promoter region and up-regulated its expression level (**Figure 2J**). These results indicate that radiation and Santacruzamate A can inhibit the activity of HDAC2 and up-regulate the level of histone acetylation of the MIR22HG promoter region to increase the expression of MIR22HG.

### MIR22HG Can Increase the Radiosensitivity in HCC *via* miR-22-5p

**Figure 3:**
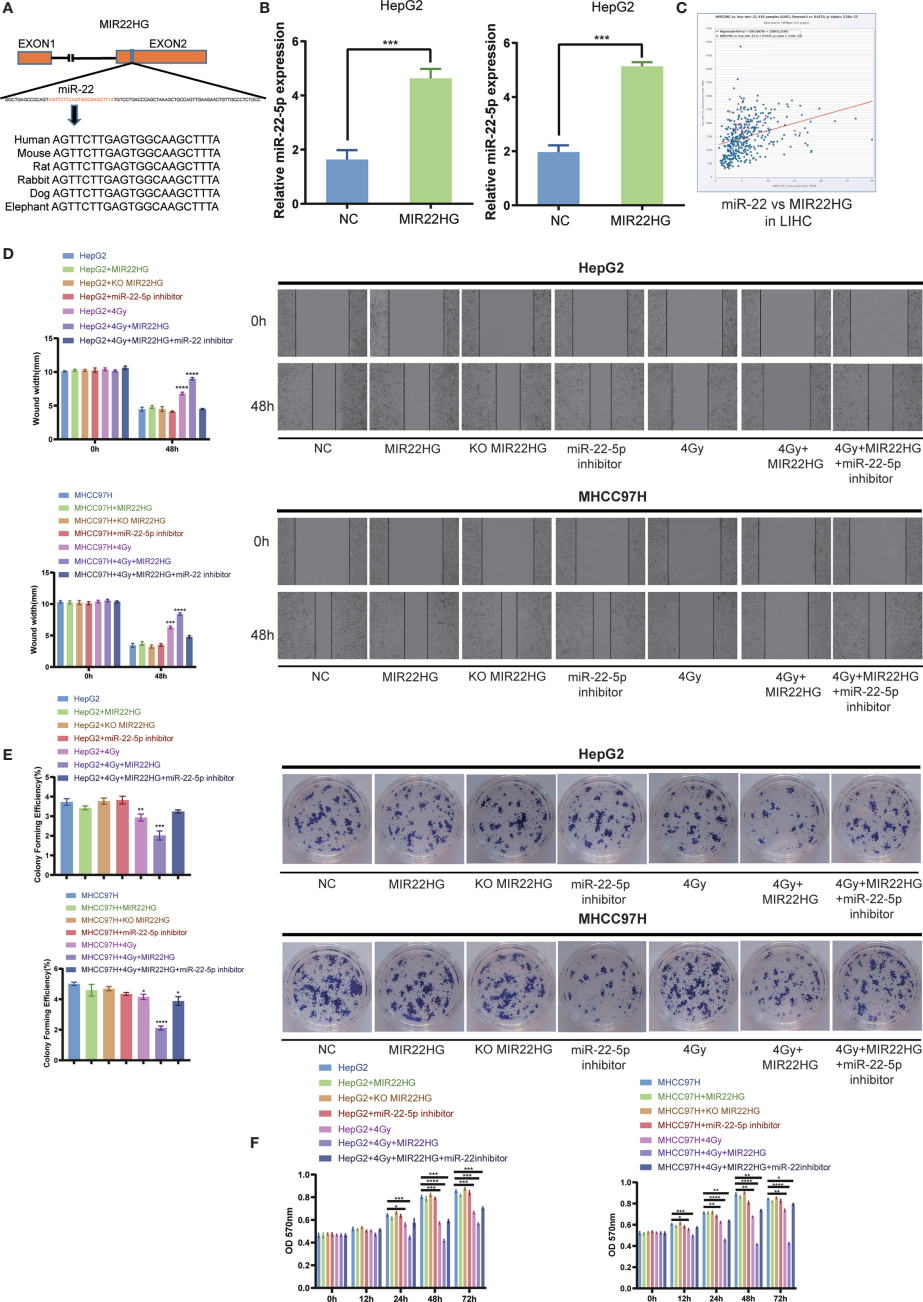
**(A)** Human miR-22 is located in exon 2 of *MIR22HG*. miR-22-5p is conserved between humans, mice, rats, rabbits, dogs, and elephants. **(B)** The miR-22-5p expression level was detected in MIR22HG-overexpressing HepG2 and MHCC97H cells using qRT-PCR. **(C)** Correlation of MIR22HG and miR-22-5p expression in TCGA cohorts. **(D)** Wound healing assay showing the migration of HepG2 and MHCC97H cells with different treatments. *Compared with the HepG2 and MHCC97H groups. **(E)** Colony formation of HepG2 and MHCC97H cells with different treatments. *Compared with the HepG2 and MHCC97H groups. **(F)** MTT assay showing the cell proliferation of HepG2 and MHCC97H cells with different treatments. Mean ± SD (n = 3 independent experiments). *p < 0.05, **p < 0.01, ***p < 0.001, ****p < 0.0001.

The high degree of conservation of the miR-22-containing region of MIR22HG among mammals implies an important role for miR-22-5p (**Figure 3A** **)**. Overexpression of MIR22HG in HepG2 and MHCC97H up-regulated miR-22-5p expression (**Figure 3B** **)**. Analysis of TCGA database showed that MIR22HG was positively correlated with miR-22-5p expression in liver cancer (**Figure 3C** **)**. The results of the wound healing assay showed that MIR22HG increased the inhibition of migration *via* miR-22-5p (**Figure 3D** **).** The results of the colony formation assay showed that MIR22HG increased the inhibition of proliferation of HCC cells *via* miR-22-5p (**Figure 3E** **).** The MTT assay showed that MIR22HG increased the lethality of radiation in HCC cells *via* miR-22-5p (**Figure 3F** **)**. Therefore, our results show that MIR22HG can increase the radiosensitivity of HCC *via* miR-22-5p.

### Santacruzamate A Increases Radiosensitivity Through the Up-regulation of MIR22HG Expression by Increasing the Level of Histone Acetylation

**Figure 4:**
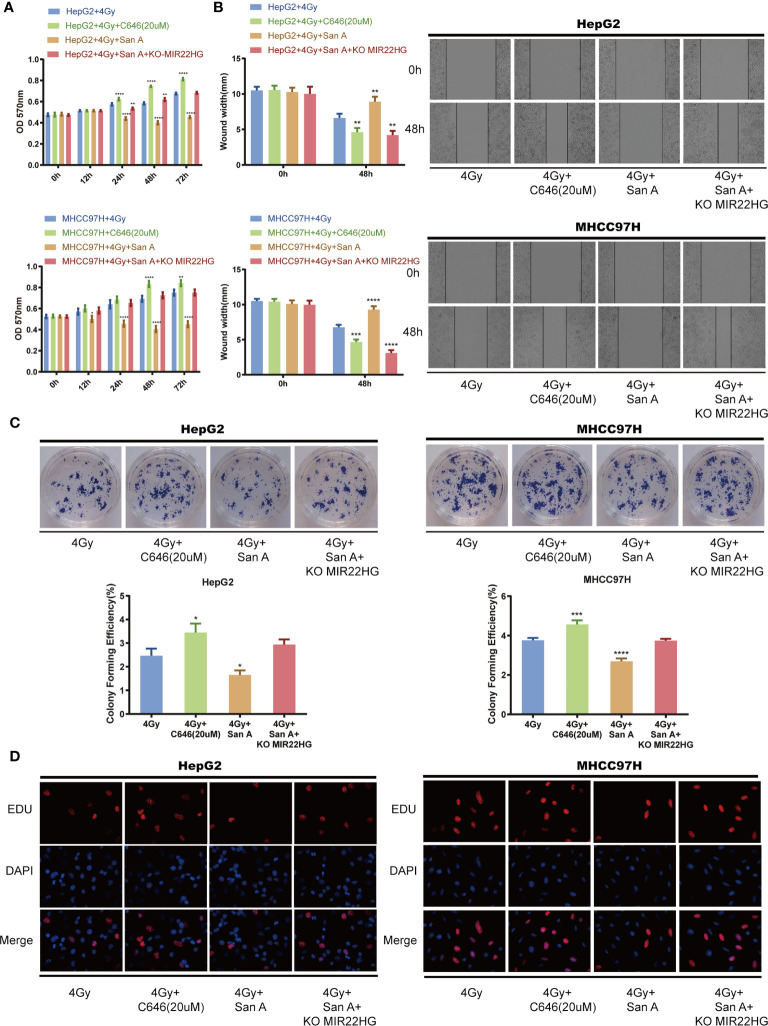
**(A)** MTT assay showing the cell proliferation of HepG2 and MHCC97H cells with different treatments. *Compared with HepG2 + 4 Gy and MHCC97H + 4 Gy groups. **(B)** Wound healing assay showing the migration of HepG2 and MHCC97H cells with different treatments. *Compared with HepG2 + 4 Gy and MHCC97H + 4 Gy groups. **(C)** Colony formation of HepG2 and MHCC97H cells with different treatments. *Compared with HepG2 + 4 Gy and MHCC97H + 4 Gy groups. **(D)** EdU assay showing the cell proliferation of HepG2 and MHCC97H cells with different treatments. Mean ± SD (n = 3 independent experiments). *p < 0.05, **p < 0.01, ***p < 0.001, ****p < 0.0001. San A, Santacruzamate A; KO MIR22HG, knockout MIR22HG.

Cells were treated with 4 Gy of radiation in the HCC cells HepG2 and MHCC97H, and 20 µM of C646 inhibited the histone acetylation levels, and Santacruzamate A inhibited the histone deacetylation levels. The MTT assay showed that C646 can reduce the lethal rate of radiation on HCC cells, whereas Santacruzamate A can increase the lethality of radiation in tumor cells. MIR22HG knockout antagonized the radiosensitization effect of Santacruzamate A (**Figure 4A** **).** The results of the wound healing assay showed that C646 can reduce the inhibitory effect of radiation in the migration ability of HCC cells, and Santacruzamate A can increase the inhibitory effect of radiation in the migration ability of HCC cells. MIR22HG knockdown antagonized the function of Santacruzamate A (**Figure 4B** **)**. The results of the colony formation assay showed that C646 can decrease the inhibitory effect of radiation in the proliferation of HCC cells, whereas Santacruzamate A increases the inhibitory effect of radiation in the proliferation of HCC cells. MIR22HG knockout antagonized the effect of Santacruzamate A (**Figure 4C** **)**. The results of EdU showed that the fluorescence intensity of the radiation + C646 group was enhanced, the cell proliferation ability was stronger than that of the radiation group, and Santacruzamate A increased the inhibition of proliferation of HCC by radiation, and MIR22HG knockout antagonized the effect of Santacruzamate A (**Figure 4D** **)**. Our results indicate that Santacruzamate A increases the radiosensitivity of HCC cells through the up-regulation of MIR22HG expression by increasing histone acetylation levels.

### *In Vivo* Experiments Further Demonstrate That Increasing Histone Acetylation Levels Promotes Radiosensitivity by Up-regulating MIR22HG Expression

**Figure 5:**
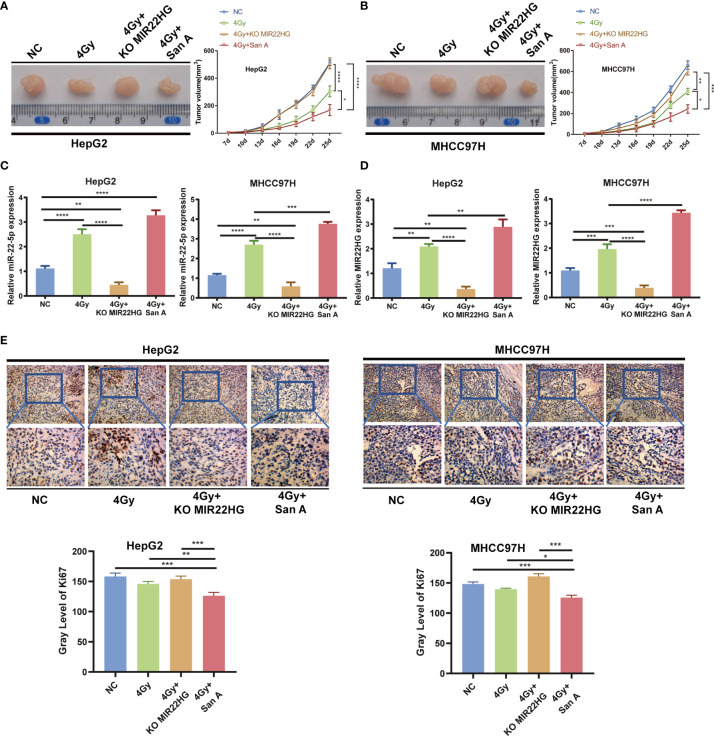
**(A, B)** Tumor tissues isolated from the indicated mice on day 25 post-transplant. Tumor growth curve in nude mice. After tumor cells were injected subcutaneously into the right flank of the abdomen of the nude mice, the short and long diameters of the tumors were measured every 3 days and tumor volumes (mm^3^) were calculated. **(C, D)** qPCR of *MIR22HG* and miR-22-5p mRNA expression in HepG2 and MHCC97H xenografts. **(E)** Immunohistochemistry showing the Ki67 expression of HepG2 and MHCC97H xenografts. Mean ± SD (n = 3 independent experiments). *p < 0.05, **p < 0.01, ***p < 0.001, ****p < 0.0001.

The above results indicate that Santacruzamate A can increase the radiosensitivity of HCC cells by up-regulating histone acetylation to increase the expression level of MIR22HG. To further confirm whether Santacruzamate A and MIR22HG affect the radiosensitivity of liver cancer *in vivo*, we established the NC group and radiation 4 Gy only treated group, radiation 4 Gy + Santacruzamate A group, and radiation 4 Gy + Santacruzamate A + MIR22HG knockout group. The corresponding cell suspensions were inoculated subcutaneously in nude mice. The mice in the experimental group were irradiated with 4 Gy radiation and the tumor volume was calculated. After 5 weeks, the mice were euthanized and the tumors were collected for analysis. The results showed that in the radiation 4 Gy group, the tumor volume was smaller than that of the control group, and the tumor volume of the 4 Gy + Santacruzamate A group was smaller than that of the other three groups, while the radiation 4 Gy + Santacruzamate A + MIR22HG knockout group showed no significant difference compared to that of the control group (**Figures 5A, B** **)**. PCR showed that the expression levels of MIR22HG and miR-22-5p in the 4 Gy + Santacruzamate A + MIR22HG knockout group were not significantly different from those in the control group, whereas the expression levels of MIR22HG and miR-22-5p in the 4 Gy and 4 Gy + Santacruzamate group A were significantly higher than those in the control group (**Figures 5C, D** **)**. The results of IHC showed that the expression level of Ki67 in the 4 Gy + Santacruzamate A + MIR22HG knockout group was not significantly different from that in the control group, whereas the expression level of Ki67 in the radiation 4 Gy group and the radiation 4 Gy + Santacruzamate A group was significantly lower than that in the control group (**Figure 5E** **).** These data indicate that Santacruzamate A increases radiosensitivity through the up-regulation of MIR22HG expression by increasing the level of histone acetylation *in vivo*.

## Discussion

This study is the first, to our knowledge, to explore the relationship between MIR22HG and the radiosensitivity of HCC. The results showed that radiation could up-regulate MIR22HG and miR-22-5p expression and inhibit HDAC2 activity. The decrease in HDAC2 activity can up-regulate the histone acetylation level of the MIR22HG promoter region, thus promoting the expression of MIR22HG and miR-22-5p derived from MIR22HG. Functionally, inhibition of histone deacetylation of the MIR22HG promoter can up-regulate MIR22HG expression to increase the expression level of miR-22-5p, thus promoting the radiosensitivity of HCC. Therefore, this study is likely to provide a new target for the radiosensitiaztion of liver cancer.

The HDAC family plays an important role in tumor development, chemosensitivity, and radiosensitivity (26–29). The HDAC family can affect gene transcription and function by influencing the level of histone acetylation of promoter regions. In prostate cancer, for example, HDAC3 interacts with the SP1 site on the miR-451 promoter, increasing the miR-451 promoter histone deacetylation to inhibit miR-451 expression (30). Sun et al. found that hypoxia can promote HDAC3 expression, thus inducing the histone deacetylation of the lncRNA LET promoter, and inhibit the expression of lncRNA LET (23). In addition, HDAC4 inhibits the miR-200a expression by interacting with the SP1 site in the promoter region of miR-200a in HCC (31). These results suggest that under appropriate conditions, the activity of HDAC family members can be altered to induce histone deacetylation in the promoter region. In this study, we found that radiation inhibits histone HDAC2 expression and increases the level of histone acetylation in the promoter region of MIR22HG, thus, increasing the expression levels of the tumor suppressor genes MIR22HG and miR-22-5p. To date, no related studies have been found for HCC, and we discovered that this mechanism can increase HCC radiosensitivity.

There is a complex relationship between miRNAs and their host genes, such as MIR17HG, and its source miR-17-92 cluster can amplify B-cell receptor signaling *via* the inhibition of ITIM proteins (32). This mechanism may be a novel lymphomagenic feed-forward loop. miR-11 directly inhibits the expression of RPR and HID in *Drosophila melanogaster*, and the transcription of *RPR* and *HID* in *D. melanogaster* is up-regulated by the miR-11 host gene *dE2F1* (33). Liu and Wu previously found that miR-22-3p and its host gene MIR22HG are down-regulated in HCC tissues and play a role as tumor suppressor genes (16). In this study, we found that the expression of miR-22-5p and MIR22HG was down-regulated in HCC tissues, which was consistent with the findings of Liu et al. and previous findings in leukemia and breast cancer. We also explored the reasons for the further decrease in MIR22HG expression.

>miR-22-5p expression was altered by the overexpression or knockout of the *MIR22HG* gene, which suggests that they are functionally related. We found that MIR22HG increased the radiosensitivity of HCC cells by up-regulating miR-22-5p expression, whereas inhibiting miR-22-5p decreased the radiosensitivity of HCC cells. Our results showed that MIR22HG and miR-22-5p are co-expressed and functionally consistent.

Overall, our results suggest that radiation can inhibit HDAC2 expression, and that the down-regulation of HDAC2 can increase the histone acetylation of the MIR22HG promoter region, thus, increasing the expression of MIR22HG, and thereby increasing the radiosensitivity of HCC through the production of miR-22-5p. This finding will aid in the identification of new targets for radiotherapy treatments for liver cancer.

## Conclusion

The inhibition of HDAC2 expression promotes histone acetylation in the MIR22HG promoter region, thereby up-regulating MIR22HG expression and promoting the production of miR-22-5p, ultimately increasing the sensitivity of liver cancer radiotherapy. Our findings provide new ideas for the further development of treatments for HCC.

## Data Availability Statement

The raw data supporting the conclusions of this article will be made available by the authors, without undue reservation.

## Ethics Statement

The animal study was reviewed and approved by The IRB of Third Xiangya Hospital, Central South University.

## Author Contributions

QJ conducted the PCR, WB, and all functional experiments, and wrote the manuscript. HH conducted the animal experiments. SY and LJ completed the activity detection of HDAC. YPa, XL, and YPe contributed to the experiments and analyzed the data. All authors contributed to the article and approved the submitted version.

## Funding

This work was supported by the National Natural Science Foundation of China (81872473) and the Key R&D projects of the Hunan Science and Technology Planning Project (2017SK2052).

## Conflict of Interest

The authors declare that the research was conducted in the absence of any commercial or financial relationships that could be construed as a potential conflict of interest.

## Abbreviations

HCC, hepatocellular carcinoma; lncRNA, long non-coding RNA; MIR22HG, microRNA 22 host gene; HDAC2, histone deacetylase 2; ChIP, chromatin immunoprecipitation; PCR, polymerase chain reaction; WB, Western blot; EdU, 5-ethynyl-2′-deoxyuridine.
