# Supplementary material for: lncRNA MIR22HG-Derived miR-22-5p Enhances the Radiosensitivity of Hepatocellular Carcinoma by Increasing Histone Acetylation Through the Inhibition of HDAC2 Activity
Source: Front Oncol. 2021 Feb 24;11:572585. doi: 10.3389/fonc.2021.572585 (PMC7943860; doi:10.3389/fonc.2021.572585)
Supplement: Supplementary file 1 [file DataSheet_1.docx]

**Supplementary Figure legend**

**Supplementary Figure. (A, B)** The activity of HDAC1 and HDAC3 in HCC cells Huh7, HepG2, Hep3B, BEL-7402, MHCC97H, MHCC97L, and human normal liver cell L02. * compare with L02 group. **(C)** The activity of HDAC1 in HepG2 and MHCC97H cells treated with different dose of irradiaton. * compare with HepG2 and MHCC97H group. **(D)** The activity of HDAC3 in HepG2 and MHCC97H cells treated with different dose of irradiaton. * compare with HepG2 and MHCC97H group. Mean ± SD (n = 3 independent experiments). * *p*＜0.05, ** *p*＜0.01, *** *p*＜0.001, **** *p*＜0.0001
