# Supplementary figures and images for: lncRNA MIR22HG-Derived miR-22-5p Enhances the Radiosensitivity of Hepatocellular Carcinoma by Increasing Histone Acetylation Through the Inhibition of HDAC2 Activity
Source: Front Oncol. 2021 Feb 24;11:572585. doi: 10.3389/fonc.2021.572585 (PMC7943860; doi:10.3389/fonc.2021.572585)

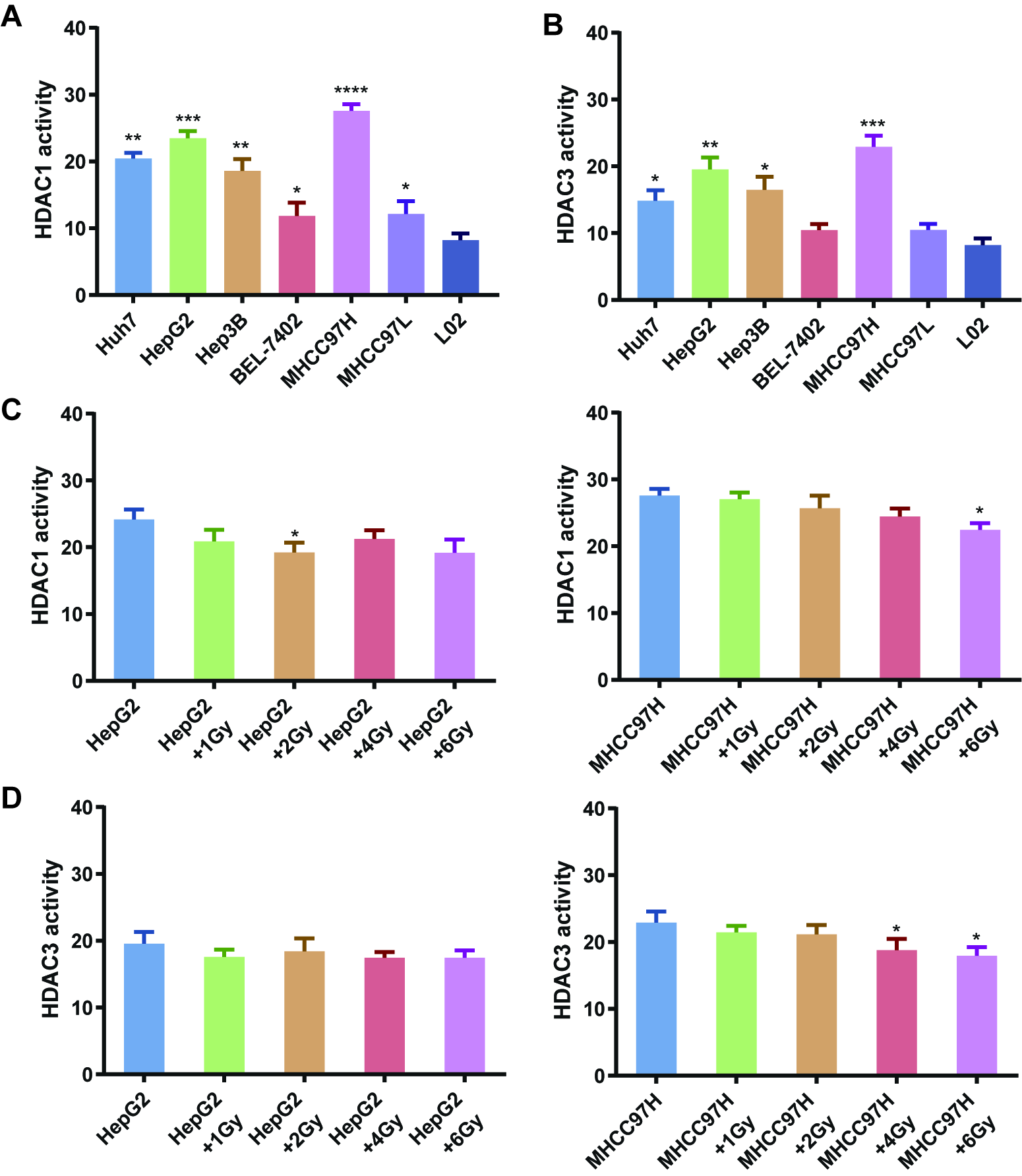

Supplement: Supplementary file 2 [file Image_1.tif]
